# Mechanical Properties of Fully Recycled Aggregate Concrete Reinforced with Steel Fiber and Polypropylene Fiber

**DOI:** 10.3390/ma17051156

**Published:** 2024-03-01

**Authors:** Lijuan Zhang, Xiang Li, Changbin Li, Jun Zhao, Shengzhao Cheng

**Affiliations:** 1 School of Mechanics and Safety Engineering, Zhengzhou University, No. 100 Science Avenue, Zhengzhou 450001, China; zhanglj0526@zzu.edu.cn (L.Z.); 15937638030@163.com (X.L.); lcb5009@163.com (C.L.); zhaoj@zzu.edu.cn (J.Z.); 2 China Construction Seventh Engineering Division, Co., Ltd., Zhengzhou 450004, China

**Keywords:** fully recycled aggregate concrete, mechanical properties, polypropylene fiber, steel fiber, load–displacement curve, toughness

## Abstract

The study and utilization of fully recycled aggregate concrete (FRAC), in which coarse and fine aggregates are completely replaced by recycled aggregates, are of great significance in improving the recycling rate of construction waste, reducing the carbon emission of construction materials, and alleviating the ecological degradation problems currently faced. In this paper, investigations were carried out to study the effects of steel fiber (0.5%, 1.0%, and 1.5%) and polypropylene fiber (0.9 kg/m^3^, 1.2 kg/m^3^ and 1.5 kg/m^3^) on the properties of FRAC, including compressive strength, splitting tensile strength, the splitting tensile load–displacement curve, the tensile toughness index, flexural strength, the load–deflection curve, and the flexural toughness index. The results show that the compressive strength, splitting tensile strength, and flexural strength of fiber-reinforced FRAC were remarkably enhanced compared with those of ordinary FRAC, and the maximum increase was 56.9%, 113.3%, and 217.0%, respectively. Overall, the enhancement effect of hybrid steel–polypropylene fiber is more significant than single-mixed fiber. Moreover, the enhancement of the crack resistance, tensile toughness, and flexural toughness obtained by adding steel fiber to the FRAC is more significant than that obtained by adding polypropylene fiber. Furthermore, adding polypropylene fiber alone and mixing it with steel fiber showed different FRAC splitting tensile and flexural properties.

## 1. Introduction

With the rapid development of the construction industry in recent years, concrete structural buildings using natural resources as raw materials have been exposed to many drawbacks, such as high resource and energy consumption, poor environmental benefits, etc. Recycled aggregate concrete (RAC) is a kind of concrete made by replacing natural aggregates with appropriately sized recycled aggregates obtained through the crushing, cleaning, and grading of construction waste. The research and utilization of recycled concrete can not only solve the problem of the difficult disposal of large amounts of construction waste but can also reduce the demand for natural aggregate resources in the construction industry and reduce the damage to the ecological environment caused by the production of aggregates [1,2,3]. Furthermore, it is estimated that using recycled aggregates instead of natural aggregates for concrete construction could save 10–20% of the material cost [4]. In the last few decades, recycled aggregate concrete made from different types of construction waste aggregates, such as waste concrete, rubber, plastics, ceramics, bricks, glass, ceramic tiles, and so on, has been investigated [5,6,7,8,9,10]. And most of the research focuses on the RAC with waste concrete as a coarse aggregate because the proportion of waste concrete in construction waste is the highest. The research results show that waste concrete aggregate not only contains the original aggregate but also contains mortar solidified on the surface of the aggregate, and the presence of this mortar will make the crushing index and porosity of the recycled aggregate much larger than that of the natural aggregate. Therefore, the strength and durability of recycled concrete under the same mix ratio are much lower than those of a natural aggregate concrete, which greatly limits the application of recycled concrete [11,12,13,14].

In recent years, researchers have used various kinds of fiber to make up for the inherent defects of recycled concrete and improve the mechanical properties of RAC. Carneiro et al. [15] studied the compressive stress–strain behavior of recycled concrete with natural coarse and fine aggregates replaced by recycled coarse fine aggregates at two levels, 0% and 25%, by volume. The results showed that steel fiber (SF) could effectively improve the brittleness of RAC and control the post-crack regime of the stress–strain curve of the recycled concrete mixtures, making their behavior under compression similar to that of fiber-reinforced natural aggregate concrete (NAC). Afroughsabet [16] added hooked steel fibers with a fiber volume content of 1% to reinforce recycled concrete with different recycled coarse aggregate substitution rates (50% and 100%) and found an up-to-60% increase in splitting tensile strength and an up-to-88% increase in flexural strength at 28 days. The research of Wang et al. [17] shows that the static elastic modulus of polypropylene fiber-reinforced fully recycled coarse aggregate concrete decreases with the growth of the fiber volume fraction and length–diameter ratio, while the compressive strength and equivalent compressive toughness index initially increases then declines. Ye et al. [18] tested the compressive and flexural properties of fully recycled coarse aggregate concrete with polypropylene fiber (PF) and found that with the increase in the volume fraction of PF, the flexural strength increased continuously, while the ratio of axial compression toughness first increased and then decreased as a whole. Moreover, they concluded that the physical and mechanical properties of FRCA concrete were better when the volume ratio of PF was 0.2%. Das et al. [19] found that PF played a significant role in crack bridging within the concrete. However, when the fiber volume fraction was high, voids tended to be created between the cement paste and the fibers, reducing the concrete’s strength. Only a suitable PF admixture could better enhance the matrix crack resistance. Since single fiber incorporation only improves the performance of concrete at a single scale, many scholars have incorporated fibers of different sizes and properties into concrete in a particular proportion, leveraging their respective advantages to form complementary and synergistic effects, thereby improving the comprehensive performance of the matrix [20,21,22,23,24]. He et al. [25] showed that the combination of steel fiber and polypropylene fiber exhibited excellent coupling effects, significantly improving the strength and bending toughness of fully recycled coarse concrete. Mohseni et al. [26] reported that when the natural coarse aggregate was replaced by 20% recycled coarse aggregate, hybrid steel–polypropylene fiber could improve the compressive strength and water absorption resistance of recycled concrete, and the splitting tensile strength of RAC increased notably with the increase in the total fiber dosage. Meanwhile, the crack width at the fibers–matrix interface was smaller than that at the aggregate–cement interface in the microscope test, and the microstructure of the cement slurry around the fibers was denser. Sharma and Senthil [27] noted that steel fiber and polypropylene fiber hybridization led to higher stiffness, static modulus of elasticity, and flexural strength due to the strain-hardening response in concrete with recycled coarse aggregate (25% replacement). Currently, there is limited research on the effect of fiber on fully recycled concrete. Ding et al. [28] reported that the incorporation of PF negatively affected the compressive strength and splitting tensile strength of fully recycled concrete but enhanced the flexural strength and fracture properties. Li et al. [29] observed that basalt fiber improved the bonding performance in recycled aggregate, made the interface strength higher, and enhanced the shear failure of fully recycled concrete when the load grade reached 70%.

In summary, the current research on fiber-reinforced recycled concrete has mainly focused on the performance analysis of recycled coarse aggregate concrete (using only recycled coarse aggregate to replace natural coarse aggregate). However, fully recycled aggregate concrete (wherein both natural coarse and fine aggregates are completely replaced by recycled aggregate) can significantly improve building materials’ recycling and reuse rate. Compared to recycled coarse aggregate concrete, it can more effectively alleviate the problems of ecological environment deterioration and natural resource consumption. Therefore, in this paper, 16 groups of specimens, including polypropylene-fiber-reinforced fully recycled aggregate concrete (PFRFRAC), steel-fiber-reinforced fully recycled aggregate concrete (SFRFRAC), hybrid steel–polypropylene-fiber-reinforced fully recycled aggregate concrete (HFRFRAC), and ordinary fully recycled aggregate concrete, were designed to conduct compressive, splitting tensile, and flexural tests to investigate the influence of polypropylene fiber, steel fiber, and hybrid steel–polypropylene fiber on the mechanical properties of the fully recycled aggregate concrete to provide a reference for the future engineering application of fully recycled aggregate concrete.

## 2. Experiment

### 2.1. Materials

**Figure 1:**
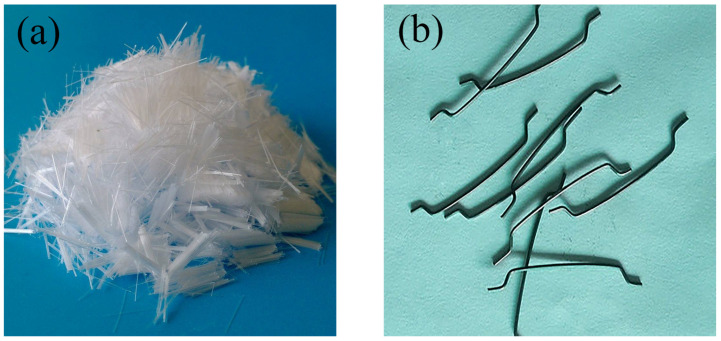
Fiber features: (**a**) polypropylene fiber; (**b**) steel fiber.

P.O 42.5 ordinary silicate cement produced in China was used in the experiments. Its relevant performance indexes are shown in Table 1. F-type fly ash from power plants was used as an admixture to partially replace cement. The main performance indexes of fly ash are shown in Table 2. And the chemical composition of cement and fly ash are given in Table 3. The recycled sand produced by Zhengzhou Gujia Technology Industry Co., Ltd. (Zhengzhou, China), was used as the recycled fine aggregate, with a fineness modulus of 3.3, an apparent density of 2570 kg/m^3^, and a bulk density of 356 kg/m^3^. The sand’s gradation is shown in Table 4. The raw material of recycled coarse aggregate (RCA) was taken from the waste concrete after the demolition of Zhengzhou Rainbow Bridge, which is 5~20 mm gravel after crushing and sifting. The main performance indexes and gradation of RCA are listed in Table 5 and Table 6, respectively. The steel fibers selected for the test were end-hooked steel fibers with a radius of 0.75 mm and a length of 35 mm. The polypropylene fiber was high-strength bundled monofilament fiber with a diameter of 50 μm and a length of 9 mm. Images of the two types of fiber are shown in Figure 1. The properties of steel fiber and polypropylene fiber are listed in Table 7. A polycarboxylic acid superplasticizer (SP) with a water-reducing ratio of 27% was used in the test. The water is the tap water in the Zhengzhou municipal area.

### 2.2. Mixture Proportions

Two types of fibers, steel fiber, and polypropylene fiber, were incorporated into the recycled concrete in both single and mixed ways. The content of steel fiber was taken as 0.5%, 1.0%, and 1.5%. The dosage of polypropylene fiber was taken as 0.9 kg/m^3^, 1.2 kg/m^3^, and 1.5 kg/m^3^. Because of the large volume of steel fiber, directly mixing with the external mixing method will have a worse effect on the working properties of the recycled concrete. Therefore, in this paper, steel fiber was used to replace a portion of coarse and fine aggregates by equal volume while keeping the sand rate (40%) unchanged. A total of 16 groups of tests were designed. The specific dosage of mixing ratio of each group is shown in Table 8.

### 2.3. Experiment and Methodology

The slump and mechanical property tests were conducted according to the Chinese Standards GB/T 50081-2019 [30] and JG/T 472-2015 [31]. For each concrete mixture, three cubed specimens (100 × 100 × 100 mm^3^) were used to determine the cubic compressive strength on the 28th day. The loading speed was always controlled between 0.5 and 0.8 MPa/s until the recycled concrete was damaged and recorded the compressive load.

The splitting tensile strength of each group was measured by three cubed specimens (100 × 100 × 100 mm^3^) on the 28th day, too. In order to test the transverse deformation of the recycled concrete specimen, based on the splitting tensile strength test standard, two glass slices with flat and smooth surfaces were placed diagonally before and after the concrete test block. Then, we placed a displacement meter of suitable range on the glass slice to ensure the accuracy of the deformation measurements of both the left and right sides of the specimen block. The displacement meter was placed on the glass plate to ensure the accuracy of the measurement of the deformation of both sides of the specimen. The DH3819 static collector was used to record the load and lateral deformation of the test specimen in real time through the displacement meter and load sensor, and the displacement loading rate was controlled at 0.1 mm/min. For ordinary FRAC and PFRFRAC mixtures, the specimens were loaded until they ruptured into two parts to end the test. For FRAC with steel fiber, it took a longer time to break into two parts, and the test ended after the transverse displacement of the specimen reached 3.5 mm.

Three prismatic beams (100 × 100 × 400 mm) were used to test the flexural performance of each group after 28 days of curing. The flexural performance test was conducted under a four-point flexural test, and the span of the two supports was three times the height of the beam, i.e., 300 mm. During the test, in order to reduce the test error, the test indenter should be prevented from directly contacting the casting surface of the specimen. The displacement loading rate was controlled at 0.1 mm/min, and the test was terminated when the net deflection value in the span exceeded 5 mm. The load and deflection of the tested specimen were recorded in real time by the displacement meter and load sensor. The displacement sensor was placed at the center of the span of the specimen, and the data acquisition instrument was the same as that for the splitting tensile test.

The strength growth coefficient was calculated as follows:

(1) $$\eta =f_{F}/f_{c},$$

where η is the strength growth coefficient of fiber-reinforced FRAC compared to ordinary FRAC; $f_{F}$ is the strength of ordinary FRAC; and $f_{c}$ is the strength of fiber-reinforced FRAC.

In order to compare the enhancement effect of hybrid fiber on the compressive strength of FRAC more intuitively, the hybrid effect coefficient was introduced in this paper, which was determined by the ratio of the strength growth coefficient. The hybrid effect coefficient can be expressed as follows:

(2) $$\lambda_{\mathrm{PS}}=\eta_{\mathrm{PS}}^{2}/\left(\eta_{P}\times \eta_{S}\right),$$

where $\lambda_{PS}$ is the hybrid effect coefficient of fiber FRAC strength; $\eta_{P}$, $\eta_{S}$, and $\eta_{PS}$ are the strength growth coefficients of PFRFRAC, SFRFRAC, and HFRFRAC mixtures, respectively.

When $\lambda_{PS}$ > 1, it means that there is a “positive effect” and the hybrid fiber has a better effect on recycled concrete than the single fiber; when $\lambda_{PS}$ ≤ 1, it means that there is a “negative effect” and the hybrid fiber is worse than the single fiber.

## 3. Test Results and Discussion

### 3.1. Slump

**Figure 2:**
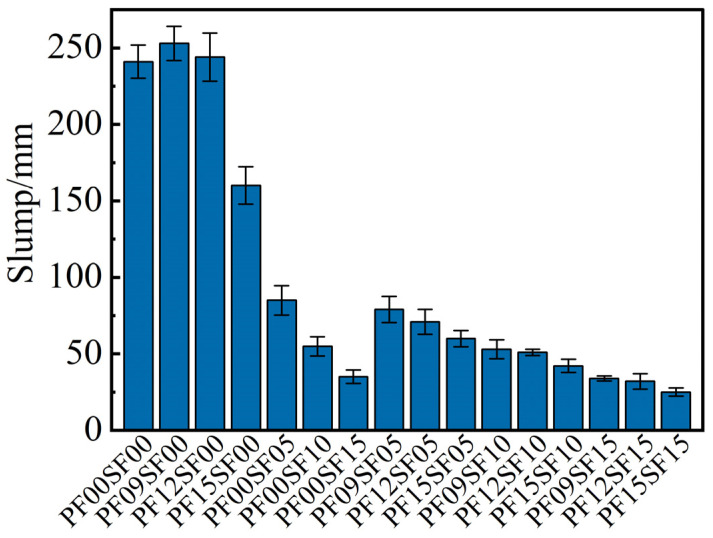
Slump results of FRAC.

The slump can reflect the workability of the fresh concrete, and the results are shown in Figure 2. Adding PF alone at the dosage of 0.9 kg/m^3^ and 1.2 kg/m^3^ had little effect on the slump of FRAC, but when the dosage was 1.5 kg/m^3^, the slump was decreased by 33.6% compared with ordinary FRAC. By contrast, the incorporation of steel fiber has a significant effect on the slump values of FRAC. As can be seen, the addition of single SF and hybrid steel–polypropylene fiber reduced the slump by approximately 64.7–85.5% and 67.2–89.6%, respectively, compared with that of ordinary FRAC.

**Figure 3:**
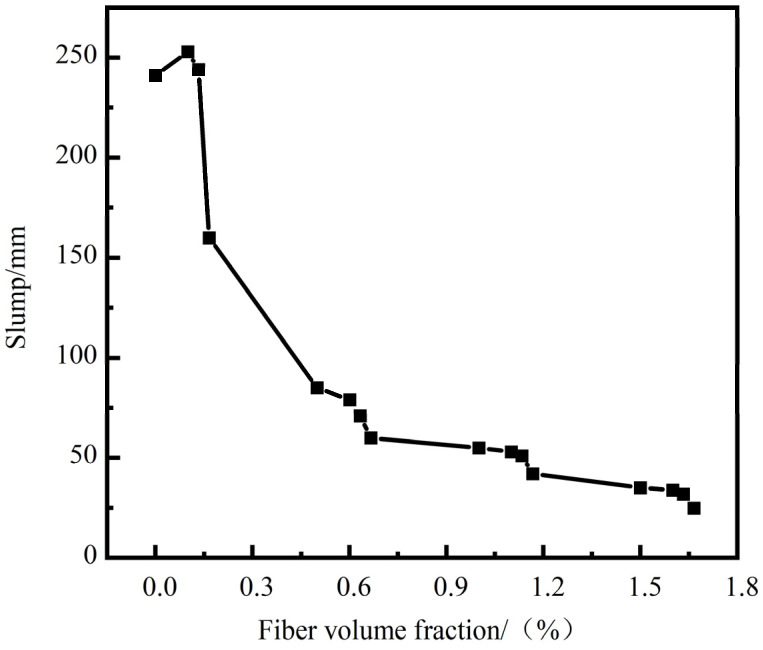
The effect of fiber volume fraction on the slump of FRAC.

Figure 3 shows the effect of fiber volume fraction on the slump value of FRAC. Overall, the slump value decreased with the increase in the total volume fraction of SF and PF. When the volume fraction of fiber was greater than 0.5%, the slump values were significantly reduced by more than 64.7% compared with those of ordinary FRAC, which might be due to the fact that the addition of fiber consumed extra water and cement paste to cover the surface of the fiber during the mixing process, increasing the roughness of the mixture and thus reducing the fluidity of the mixture [32]. Furthermore, compared with PF, the incorporation of SF could improve the adhesion between different materials of the paste and hinder the flow of the mixture, leading to a decrease in the slump value of FRAC [33]. Therefore, it is recommended to consider the influence of total fiber volume fraction on the slump value in the calculation of the unit water consumption of a concrete mixture to ensure the actual workability of FRAC.

### 3.2. Cube Compressive Strength

#### 3.2.1. Failure Morphology

**Figure 4:**
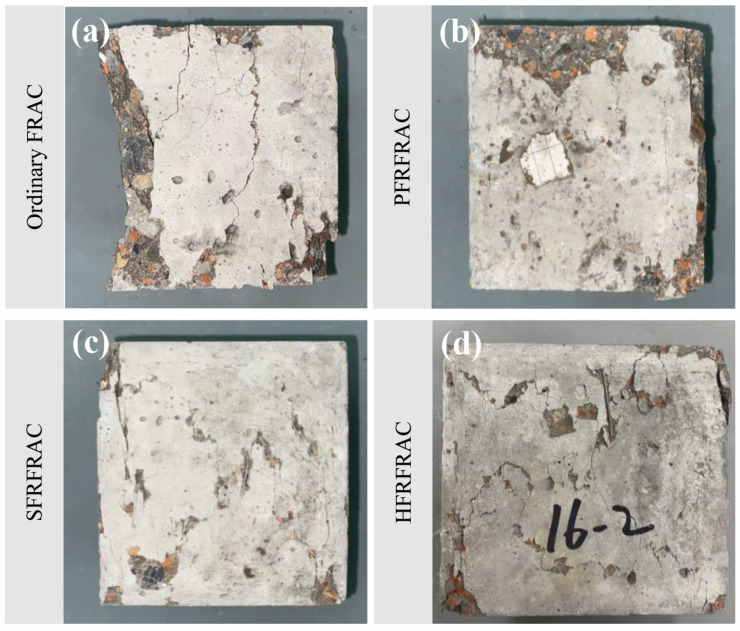
Damage morphology of four FRAC mixtures: (**a**) ordinary FRAC; (**b**) PFRFRAC; (**c**) SFRFRAC; (**d**) HFRRAC.

The comparison of the damage morphology of four FRAC mixtures is shown in Figure 4. The effect of fiber on the damage morphology of FRAC is very significant. In the compressive test of ordinary FRAC, the middle of the specimen slightly bulged outward in the elastic stage due to the hoop constraint of the bearing plate. After exceeding a certain proportion limit, the specimen underwent plastic deformation, and multiple cracks appeared soon and gradually ran through the specimen, accompanied by a large area of concrete falling off the surface. When the load exceeded the peak bearing capacity, the specimen split into several parts, and the loading force was rapidly reduced to zero. As for the PFRFRAC mixtures, the number, width, and length of cracks reduced substantially relative to the ordinary FRAC, and there was no large area of concrete peeling off from the side of the specimen. Therefore, the specimen could still ensure good integrity at the end of the compression test. As can be seen from Figure 4c,d, the damage morphology of SFRFRAC is similar to that of HFRFRAC; there was no obvious material shedding phenomenon during the compressive process, and the sound when the steel fiber was disconnected could be heard. Furthermore, most of the cracks appeared in the surface area of the specimen and did not extend to the whole specimen. After the load reached the ultimate compressive strength capacity of concrete, the specimen showed a certain degree of ductility without sudden brittle damage, and the load was reduced to zero slowly. Although there were some fragments after the destruction, they were still attached to the specimen by fiber, and peeling it off by hand was not easy.

#### 3.2.2. Fiber Effect

**Figure 5:**
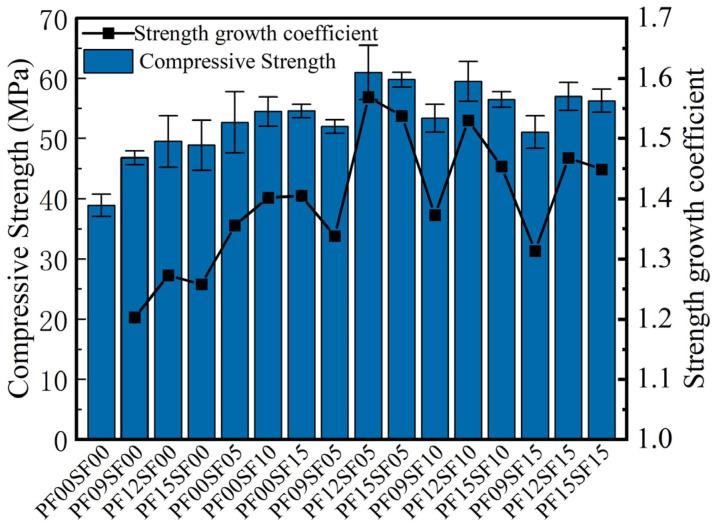
Compressive strength and strength growth coefficient of FRAC.

As seen in Figure 5, fiber has a significant effect on enhancing the compressive performance of FRAC, and the compressive strengths of all FRAC specimens with added fiber are higher than those of ordinary FRAC. The compressive strength increments of single PF- and SF-reinforced FRAC ranged from 20.3 to 27.3% and from 35.6 to 40.5%. Among them, the compressive strength of PF12SF05 was the highest, and the increase rate was 56.9%. Furthermore, the lowest increment in compressive strength was obtained for the PF09SF00.

**Figure 6:**
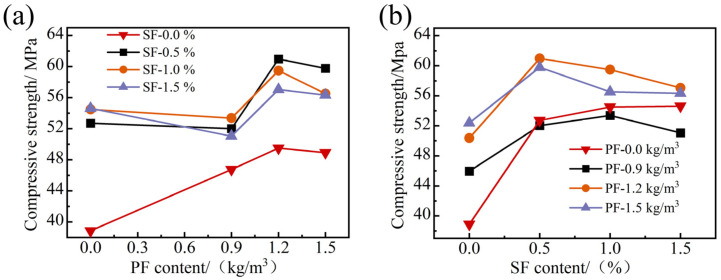
The effect of fiber content on the compressive strength of FRAC: (**a**) PF; (**b**) SF.

In Figure 6a, the compressive strength of FRAC with single polypropylene fiber increased significantly at first and then decreased slightly with the increase in polypropylene fiber content. This is because PF has the characteristics of being lightweight and small in size and can effectively fill the fine pores caused by the hydration reaction and bubble escape. When PF was moderately incorporated into FRAC, it could be uniformly distributed in the matrix in a three-dimensional manner to play a bridging role, increasing the connection strength between old concrete mortar and new concrete mortar on the surface of coarse aggregate and improving the overall compressive strength. However, when the dosage of PF was high, it could easily become intertwined in concrete, resulting in a clumping phenomenon [34,35]. Because the elastic modulus of PF was lower than that of concrete, the clumped PF caused weak compressive properties in the matrix, which was more likely to crack under compression, thus reducing the compressive strength [36]. As can be seen in Figure 6b, the enhancement effect of SF was more substantial than that of PF on FRAC compressive strength, mainly because the curved-hook SF with high tensile strength and high modulus of elasticity could undertake a portion of the tensile and compressive stresses, thus blocking the further development of microcracks within the matrix and improving the compressive performance.

**Figure 7:**
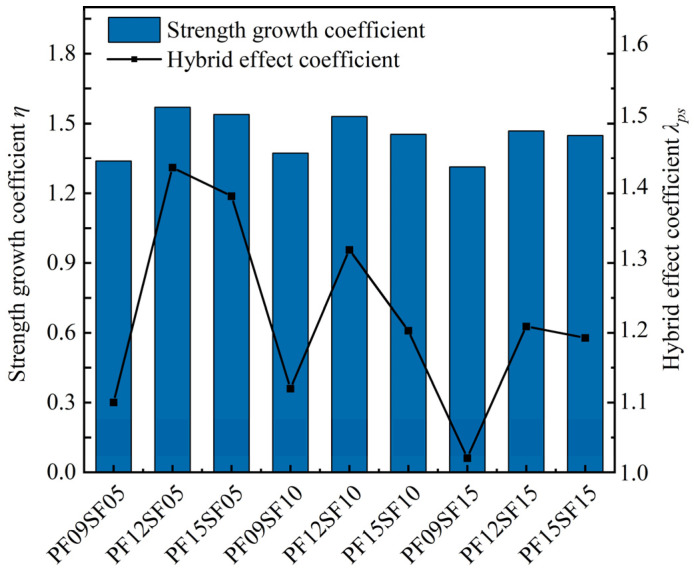
Strength growth coefficient and hybrid effect coefficient of HFRFRAC.

As regards hybrid fiber groups, when the SF content was fixed, as the PF content increased, the compressive strength of FRAC first decreased, then increased, then finally slightly decreased. When the PF dosage was unchanged, FRAC’s compressive strength increased first and then decreased with the increase in SF content. It can be seen from Figure 7 that the hybrid effect coefficients of the hybrid fiber groups are all greater than 1, indicating that the enhancement effect of hybrid fiber on the compressive strength of FRAC is better than that of the single fiber. Moreover, when PF content was 1.2 kg/m^3^ and the SF volume fraction was 0.5%, FRAC’s strength growth and hybrid effect coefficients were the highest.

The blending of SF and PF improved the compressive strength of FRAC because of their different properties. Steel fiber has a high modulus of elasticity and a large diameter, which can mainly inhibit the expansion of larger cracks; and polypropylene fiber, with a low modulus of elasticity, can inhibit the development of different types of cracks within the matrix. Specifically, PF with small volume and good adhesion improves the pore structure inside the FRAC, making the concrete denser and reducing the effect of microcracks. SF with higher tensile strength allows FRAC to act as an anchor in the matrix during compression and better restrain its transverse deformation [37]. However, when the PF content was certain, the higher the SF content, the lower the compressive strength of the recycled concrete. After blending two kinds of fiber with different moduli of elasticity, the flexible PF with a small modulus of elasticity filled the internal pores of FRAC, which affected the compactness of the concrete matrix. Due to the higher fiber dosage, some of the fiber might not be able to be dispersed uniformly [38], which further increases the internal porosity of the concrete. Moreover, SF had a large volume and high hardness and changed the internal skeleton of FRAC, which was also the main reason for the decrease in the hybrid effect coefficient when the fiber content was too large.

### 3.3. Splitting Tensile Performance

#### 3.3.1. Splitting Tensile Strength

**Figure 8:**
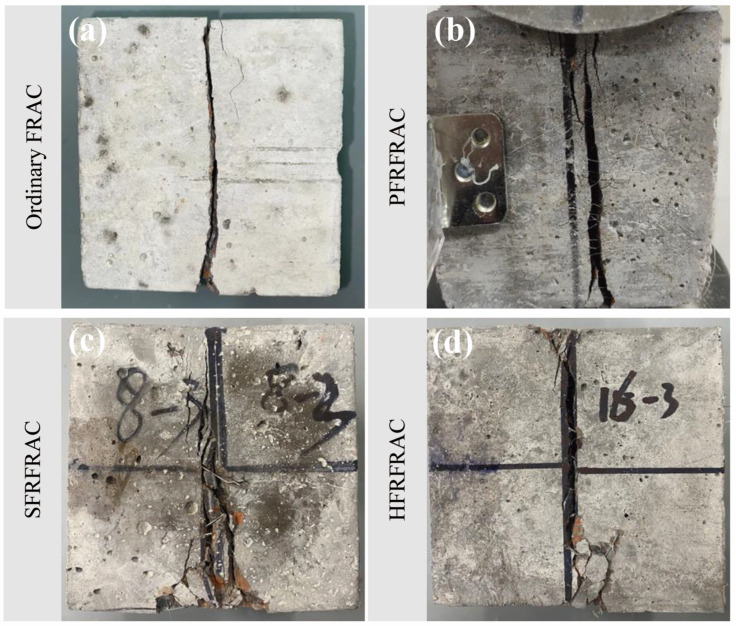
Damage morphology of four FRAC mixtures: (**a**) ordinary FRAC; (**b**) PFRFRAC; (**c**) SFRFRAC; (**d**) HFRRAC.

The comparison of the damage morphology of four FRAC mixtures in the splitting tensile test is shown in Figure 8. The damage of ordinary FRAC and PFRFRAC after the test was more serious, with cracks appearing through the specimen and the concrete splitting into two parts, whereas the FRAC containing SF was more intact after the damage; cracks appeared but the cracks did not penetrate through the specimen, and the concrete was still attached. During the test, ordinary FRAC did not show any cracks on the surface of the specimen before the peak load; when the load reached the peak, fragments began to fall off at the centerline position, and suddenly a large crack appeared, which soon extended vertically through the whole specimen, and after that, the specimen cracked into two parts. Before the PFRFRAC specimen reached the ultimate load, there were small cracks on the surface, and they expanded further with the increase in the load. After the peak load, the cracks on the surface of the specimen almost ran through the whole specimen, but some material was still connected together, and small polypropylene fibers could be seen at the cracks. PFRFRAC exhibited toughness and could withstand a certain degree of tensile stress, so the load gradually decreased after the peak load and dropped to zero when the specimen was completely divided into two parts. In comparison, the loading time of SFRFRAC and HFRFRAC mixtures was longer. When the peak load reached about 70–80%, vertical cracks first appeared at the center line of the specimen, the side of the specimen bulged out, and small fragments began to fall on the surface of the specimen. When the peak load was reached, the sound of SF disconnection could be heard, at which time the surface cracks were not obvious. After the peak load, the load decreased in a wavy manner, showing good toughness. While the lateral displacement reached 3.5 mm, SF could be seen connecting the materials together in the cracks, and the specimen maintained good integrity.

**Figure 9:**
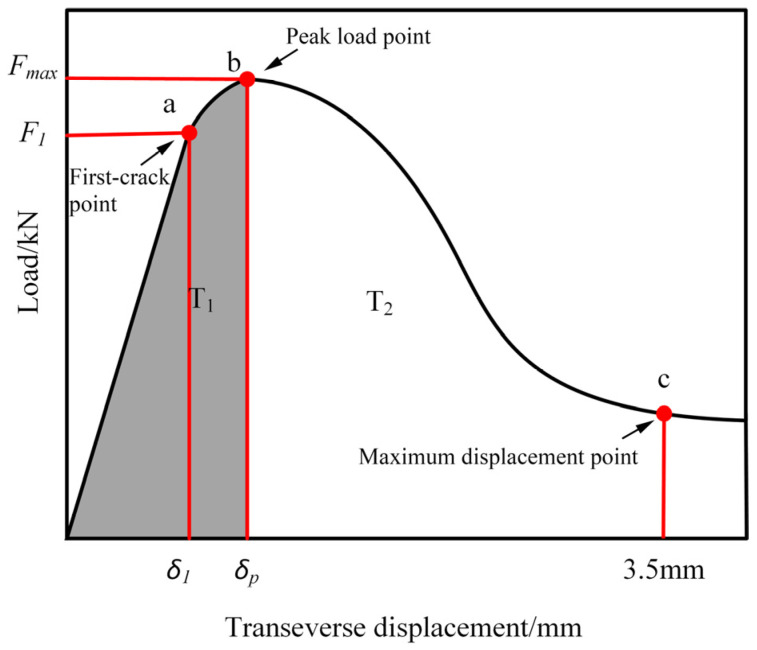
Splitting tensile load–displacement curve.

As shown in Figure 9, the splitting tensile load–displacement curve is divided into two regions, T_1_ and T_2_, where $F_{1}$ represents the crack load, $\delta_{1}$ is the crack displacement, and a-b is the crack development stage; $F_{max}$ is the peak load, $\delta_{p}$ is the peak displacement, and b-c is the failure stage of the specimen; T_1_ represents the peak toughness, which is obtained by the integral of the load–displacement curve up to the peak displacement; and the residual toughness T_2_ can be defined as the integral of the load–displacement curve from the peak displacement up to 3.5 mm.

**Figure 10:**
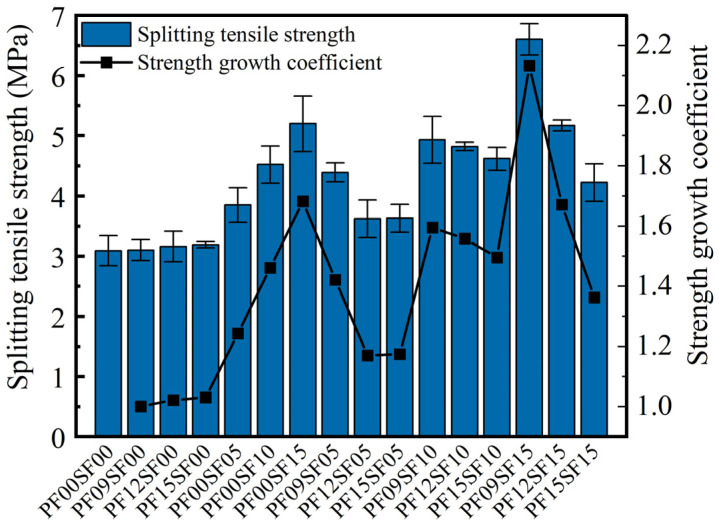
Splitting tensile strength and strength growth coefficient of FRAC.

From Figure 10, it can be seen that fiber has a significant increment effect on the splitting tensile performance of FRAC, and the splitting tensile strength of FRAC with the addition of fiber is higher than that of ordinary FRAC. Among them, the PF09SF15 group had the highest splitting tensile strength, with a growth rate of 113.3% compared to ordinary FRAC, and the PF09SF00 group had the smallest growth rate of 0.10%.

**Figure 11:**
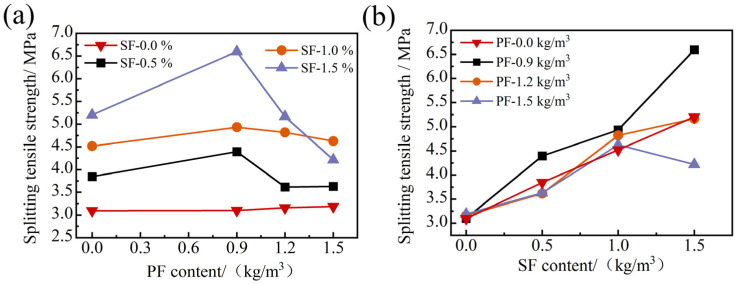
The effect of fiber content on the splitting tensile strength of FRAC: (**a**) PF; (**b**) SF.

As can be seen from Figure 11, the single PF has little influence on the splitting tensile strength of FRAC, and its enhancement effect is not as significant as that of single SF. When the PF content was 1.5 kg/m^3^, the splitting tensile strength only increased by 3.1%, while the increase reached 24.3% when the SF volume fraction was 0.5%. Since ordinary FRAC is a brittle material, damage under the influence of transverse tensile stress is easy to produce. As flexible fiber with low elastic modulus, PF can achieve specific elongation, but its tensile properties are limited, so it has little influence on the splitting tensile strength of the FRAC. As for SF, due to its irregularly curved and hooked shape, it is firmly anchored inside the FRAC, increasing the friction force between it and the matrix. Moreover, it has a large elastic modulus and high tensile strength, thus significantly improving the tensile properties of the FRAC [39].

In Figure 11, the strength change pattern of the hybrid fiber group is obvious, and the data change trend in each group is almost consistent. In Figure 11a, when SF was added and the content remained unchanged, the splitting tensile strength of the FRAC increased first and then decreased with the increase in PF content. In Figure 11b, when the PF dosage was less than 1.5 kg/m^3^, the FRAC splitting tensile strength showed a monotonic increasing trend with the increase in SF dosage. However, when the PF dosage was 1.5 kg/m^3^, the splitting tensile strength first increased and then decreased with the increase in SF dosage. In the HFRFRAC mixtures, the splitting tensile strength was the highest among the same SF dosage when the PF dosage was 0.9 kg/m^3^, and while the SF volume fraction was 0.5, 1%, and 1.5%, the increase compared to ordinary FRAC was 42.1%, 59.5%, and 113.3%, respectively.

**Figure 12:**
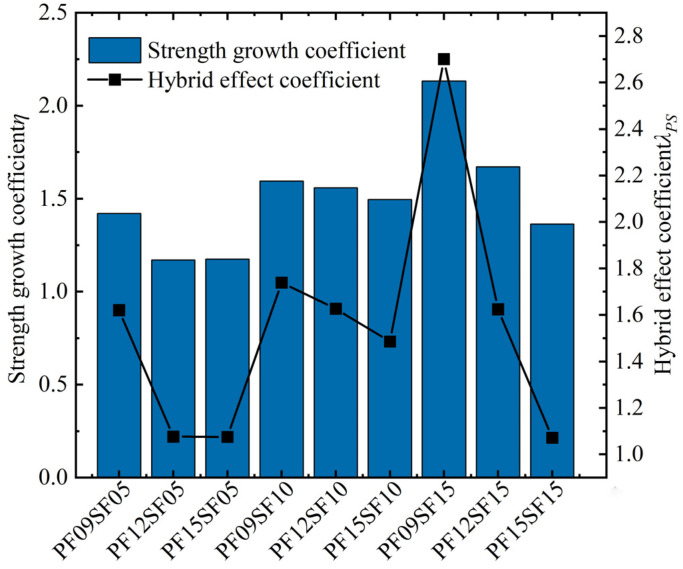
Strength growth coefficient and hybrid effect coefficient of HFRFRAC.

From Figure 12, the hybrid effect coefficients of the HFRFRAC mixtures are all greater than 1. Overall, hybrid fiber’s enhancement effect on the FRAC’s splitting tensile strength was better than that of single SF and PF. SF plays a dominant role in the splitting tensile strength of the FRAC. When the SF content was unchanged, the splitting tensile strength significantly reduced with the increase in PF content, and the higher the SF content, the greater the reduction. Relatively, when the PF dosage was unchanged, an increase in SF dosage significantly enhanced the splitting tensile strength. The strength growth coefficient and hybrid effect coefficient were greater than other hybrid fiber groups when the PF content was 0.9 kg/m^3^.

#### 3.3.2. Load–Displacement Curve

**Figure 13:**
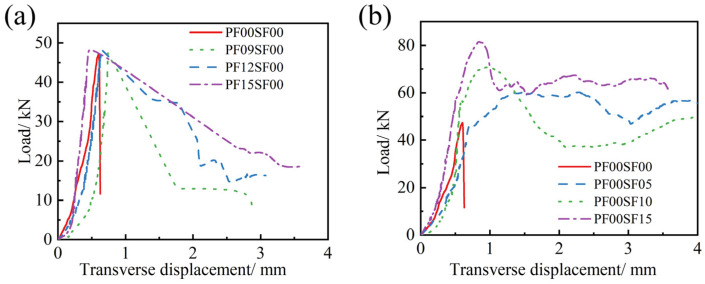
Splitting tensile load–displacement curve of FRAC with single fiber: (**a**) PF; (**b**) SF.

From Figure 13a,b, it can be seen that there is a significant difference in the effect of PF and SF on the FRAC splitting tensile load–displacement curves, with SFRFRAC having a greater peak load, more fluctuations in the curves, and a smoother descending section. The transverse displacement of SFRFRAC continued to grow smoothly when the transverse displacement was greater than 3.5 mm, whereas PFRFRAC broke into two parts before that and the test could not continue. However, it can be seen that the load–displacement curves of the two fibers are fuller than those of ordinary FRAC, indicating that both PF and SF can significantly enhance the toughness of FRAC. After cracks appeared in the ordinary FRAC specimen under tension, the load decreased sharply, and the curve showed almost no descending section. When fiber was added, the load decreased gently with the increase in transverse displacement in the failure stage. In the PF12SF00, PF15SF00, and SFRFRAC mixtures, transverse displacement increased with the load increase by a small amount in the descending section, which occurred occasionally. With the increase in fiber content, the load–displacement curves of both PFRFRAC and SFRFRAC mixtures showed an overall upward trend, and the slopes of the crack development stages increased, reflecting that the rate of crack development decreased with the increase in fiber content.

**Figure 14:**
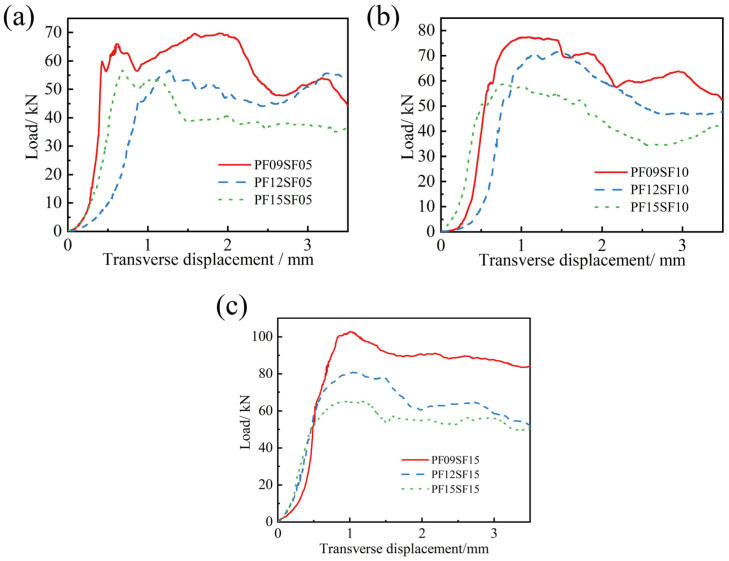
Splitting tensile load–displacement curve of HFRFRAC with different SF content: (**a**) SF-0.5%; (**b**) SF-1.0%; (**c**) SF-1.5%.

From Figure 14, it can be seen that the peak splitting tensile load decreases with the increase in PF content, and at the same time, the curve of the failure stage also shifts downward as a whole, and the slope of the crack development stage decreases accordingly. This indicated that when the PF dosage was greater than 0.9 kg/m^3^, blending with SF accelerated the crack development of FRAC, increased the brittleness of the material, and prevented SF from fully exerting its crack resistance and toughening effect, which was exactly the opposite of the situation wherein single PF incorporation increased the toughness of the FRAC. Comparison of Figure 14a–c shows that with the increase in SF volume fraction, the curves become fuller, the range of the load repeatedly rising in the curve decreases, and the slopes of the three curves at different PF dosages become closer and closer during the crack development and failure stage. In addition, when the SF content was 1.5%, the three curves were almost parallel, and the fluctuation was significantly reduced. The inflection point of the curve was caused by the sudden reduction in the resistance of the tensile section due to the withdrawal or fracture of some steel fiber, which showed that the increase in SF content notably improved the stability of the FRAC in resisting tensile failure and reduced the occurrence of sudden brittle failure in some areas of the material [40].

#### 3.3.3. Transverse Displacement

**Figure 15:**
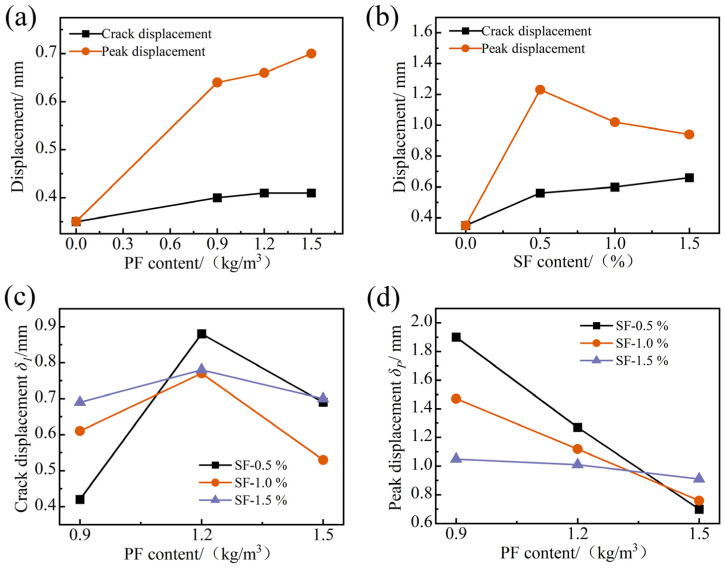
Variation of $\delta_{1}$ and $\delta_{P}$ with fiber dosage: (**a**) single PF; (**b**) single SF; (**c**,**d**) hybrid fiber.

In the concrete material structure, there is a certain relationship between the ductility and transverse deformation of the members under tensile stress. In the splitting tensile test, the transverse displacement index is an important factor affecting the tensile toughness of FRAC, and the variation pattern between transverse displacement and fiber type and content is shown in Figure 15.

After the peak load, the ordinary FRAC directly cracked into two parts, with the crack point equivalent to the peak point, and the values of $\delta_{1}$ and $\delta_{P}$ were the same. In Figure 15a, as the PF content increased from 0 to 1.5 kg/m^3^, the crack displacement $\delta_{1}$ increased from 0.35 mm to 0.41 mm with an increase of 17.1%, and the peak displacement $\delta_{P}$ increased from 0.64 mm to 0.70 mm with an increase of 10.0% when the PF content increased from 0.9 to 1.5 kg/m^3^. $\delta_{1}$ is the transverse displacement of the FRAC when the load changes suddenly and cracks first occur, and the increase in $\delta_{1}$ represents that the FRAC has an enhanced ability to resist tensile stress before cracks are produced, increasing the overall toughness before cracking. $\delta_{P}$ is the transverse displacement of the FRAC when it reaches the peak load, and the decrease in $\delta_{P}$ represents that the FRAC can better suppress further crack expansion, and its resistance to tensile deformation is enhanced during the crack development stage. It can be concluded that the crack and peak tensile deformation of FRAC is highly affected by the content of PF, and PF can effectively improve the brittleness of the matrix and slow down the occurrence time of cracks, but the inhibitory effect on crack development decreases with the increase in PF content. In Figure 15b, as the SF content increased from 0 to 1.5%, $\delta_{1}$ monotonically increased from 0.35 mm to 0.66 mm with an increase of 88.6%, and $\delta_{P}$ decreased from 1.23 mm to 0.94 mm with a decrease of 30.9% when SF content increased from 0.5 to 1.5%. This shows that the increased SF content can effectively improve the crack resistance of FRAC under tensile stress and can enhance the inhibition effect on crack expansion during the crack development stage.

Figure 15c,d show the effect of different PF and SF content on $\delta_{1}$ and $\delta_{P}$ of HFRFRAC. When the SF content was unchanged, the crack displacement and splitting tensile strength of FRAC showed the same trend of first increasing and then decreasing with the increase in PF content, and the PF content of 1.2 kg/m^3^ has the best effect on the enhancement of the crack resistance of FRAC. In Figure 15d, the $\delta_{P}$ values of FRAC with different SF dosages all showed a well-correlated monotonic decreasing trend with the increase in PF content; while in Figure 15a, the $\delta_{P}$ monotonically increased with the increase in PF content, which indicated that the addition of PF could work together with SF to strengthen the ability to suppress crack propagation in FRAC, and the bridging and crack arrest effects of fiber are more significant. In Figure 15c,d, with the increase in SF volume fraction, the variation amplitude of $\delta_{1}$ and $\delta_{P}$ obviously decreased remarkably. $\delta_{1}$ and $\delta_{P}$ changed the most when SF content was 0.5%, with the change amplitude reaching 109.5% and 171.4%, respectively, and the values of $\delta_{1}$ and $\delta_{P}$ were most stabilized when SF was 1.5%, with the change amplitude of $\delta_{1}$ and $\delta_{P}$ up to 13.0% and 15.4%, respectively. It can be shown that as the SF content increased, SF played a dominant role, and the influence of PF on the crack resistance of FRAC decreased gradually.

#### 3.3.4. Tensile Toughness

**Figure 16:**
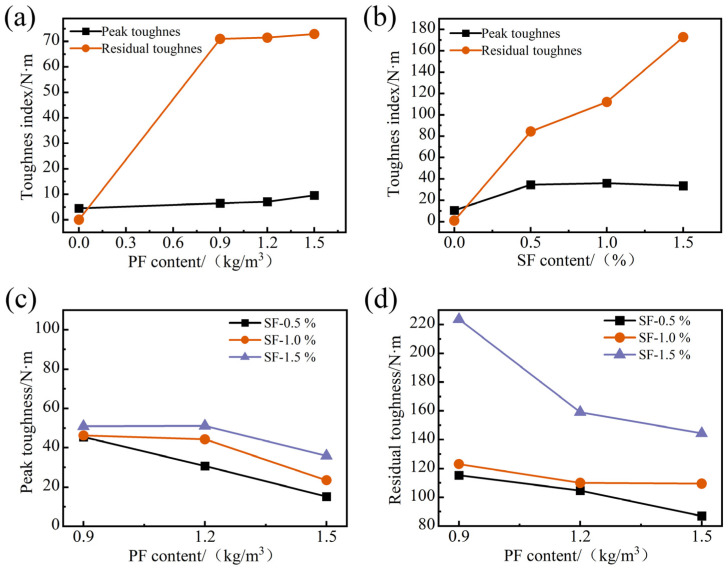
Variation of T_1_ and T_2_ with fiber dosage: (**a**) single PF; (**b**) single SF; (**c**) and (**d**) hybrid fiber.

The variation rule between the tensile toughness, fiber types, and content is shown in Figure 16. In this paper, the peak toughness T_1_ and residual toughness T_2_ are defined by the energy method. T_1_ reflects the value of external force work required for FRAC to achieve material failure, and T_2_ reflects the value of external force work required for RAC from failure to the end of the test [41].

In Figure 16a, the values of T_1_ and T_2_ increased with the increase in PF content. When the PF content increased from 0.9 kg/m^3^ to 1.5 kg/m^3^, the increase in T_2_ was only 2.66%, and the increase in T_1_ was 47.86%, which was 110.16% compared with the ordinary FRAC. Since the ordinary FRAC cracked into two parts directly after the peak load, its residual toughness T_2_ was zero, and T_2_ reached 71 N·m when the PF dosage was 0.9 kg/m^3^. However, increasing the PF dosage afterward did not significantly improve the residual toughness T_2_. From Figure 16b, it can be seen that SF has a remarkable effect on improving the toughness of FRAC. As the volume fraction of SF increased, the peak toughness of SFRFRAC did not change much, but it increased by an average of more than 6 times compared to ordinary FRAC. The residual toughness increased with the increase in SF content, and the peak toughness increased by 105.1% when the SF content increased from 0.5% to 1.5%.

In Figure 16c,d, when the PF dosage was constant, T_1_ and T_2_ increased with the increase in SF dosage, and the change of T_2_ was most significant when the SF dosage increased to 1.5%. While the SF dosage remained constant, T_1_ and T_2_ decreased with the increase in PF dosage, and the best enhancement effect on HFRFRAC was achieved when the PF dosage was 0.9 kg/m^3^. Compared to SFRFRAC mixtures with the same SF dosage, the increase in T_1_ was 31.75%, 36.89%, and 28.60%, and the increase in T_2_ was 10.0%, 60.18%, and 29.41%, respectively. However, when the PF content was 1.2 kg/m^3^ and 1.5 kg/m^3^, compared to the same SF content of the SFRFRAC mixtures, T_1_ and T_2_ had a certain degree of reduction, indicating that higher PF content had a negative impact on the tensile toughness of HFRFRAC.

### 3.4. Flexural Performance

#### 3.4.1. Peak Flexural Strength

**Figure 17:**
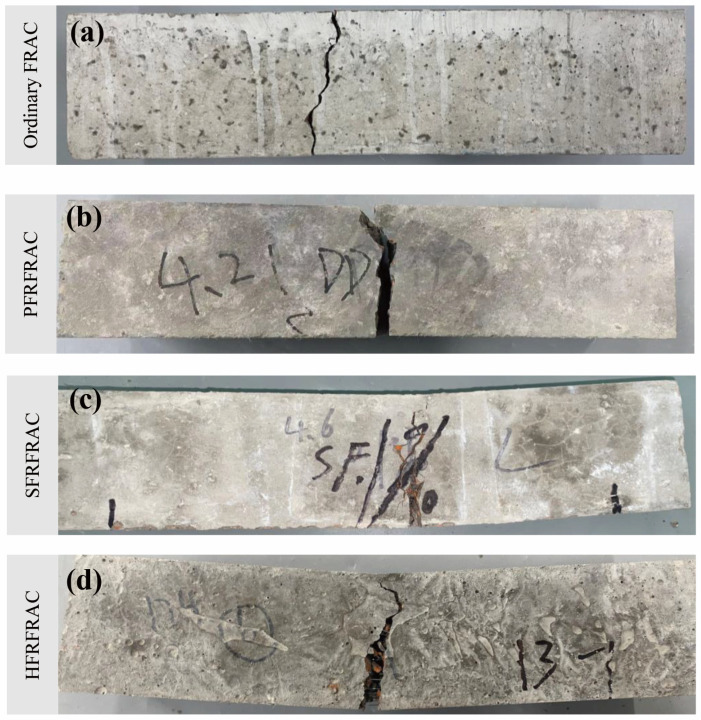
Damage morphology of four FRAC mixtures: (**a**) ordinary FRAC; (**b**) PFRFRAC; (**c**) SFRFRAC; (**d**) HFRRAC.

Fiber addition improved the flexural damage morphology of the FRAC. As shown in Figure 17, during the test, the ordinary FRAC specimens showed typical brittle damage under bending moment. When approaching the peak load, the crack first appeared at the bottom of the mid-span position, then the crack developed rapidly upward, and finally, the specimen broke into two parts with almost no ductility. The flexural damage morphology of the PFRFRAC was similar to that of the ordinary FRAC. In the initial crack, there were tiny PFs connected in the middle of the crack, which provided a certain constraint on the extension of the cracks, but the specimen also broke quickly when the ultimate failure load was reached, indicating that the toughening effect of PF was very small. The flexural damage morphology of SFRFRAC and HFRFRAC shows certain ductile characteristics. In the initial crack, the specimen first exhibited small cracks at the bottom-span center position, at which time the load decreased but the deflection value increased rapidly. After that, the load kept increasing, the bottom crack continued to extend upward, the crack width grew with the increase in the load, and part of the connecting section or broken fiber could be clearly seen at the crack. After the peak load, the load gradually decreased, the specimen could still withstand the load, and at the same time, the sound when part of the steel fiber was pulled out can be heard. The test stopped when the load dropped to 30% of the peak load, at which point the FRAC specimen still maintained its integrity.

The flexural load–deflection curves were obtained from the test, and according to the relevant provisions of Standard ASTM C1609 [42], the relevant indexes of flexural performance were calculated as shown in Table 9.

In Table 9, $f_{1}$ is the first-peak flexural strength; fp is the peak flexural strength; $\delta_{1}$ is the first-peak deflection; $\delta_{p}$ is the peak-load deflection; $f_{100}^{100}$ and $f_{150}^{100}$ are the residual flexural strengths corresponding to net deflections of l/100 (3.0 mm) and l/150 (2.0 mm) for a beam of nominal depth 100 mm, respectively. The formula is as follows:

(3) $$f=\mathrm{Fl}/bh^{2},$$

where f is the flexural strength (MPa); b is the section width of the specimen, which is 100 mm in this paper; h is the section height of the specimen, which is 100 mm in this paper; F is the load value (N); and l is the span length between the supports, which is 300 mm in this test.

**Figure 18:**
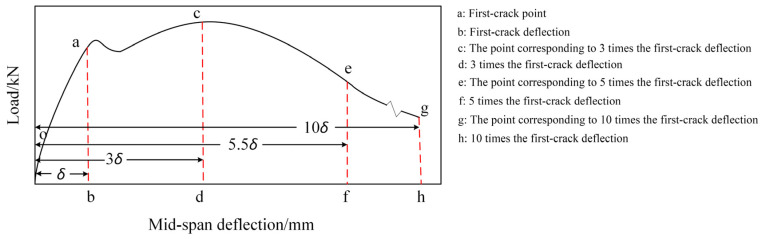
Important characteristics of the load–deflection curve.

According to the ASTM C1018 [43], the related flexural toughness indexes obtained by calculation are shown in Table 10, and the important characteristics of the load–deflection curve are shown in Figure 18.

In Table 10, toughness indexes $I_{5}$, $I_{10}$, and $I_{20}$ denote the ratios of the integral of the load–deflection curve at deflections of up to 3.0 times the first-crack deflection, 5.5 times the first-crack deflection, and 10.5 times the first-crack deflection to the integral of the curve up to first crack, respectively; $R_{5,10}$ and $R_{10,20}$ denote residual strength factors and can be expressed as follows:

(4) $$R_{5,10}=20\left(I_{10}-I_{5}\right),$$

(5) $$R_{10,20}=10\left(I_{20}-I_{10}\right).$$

**Figure 19:**
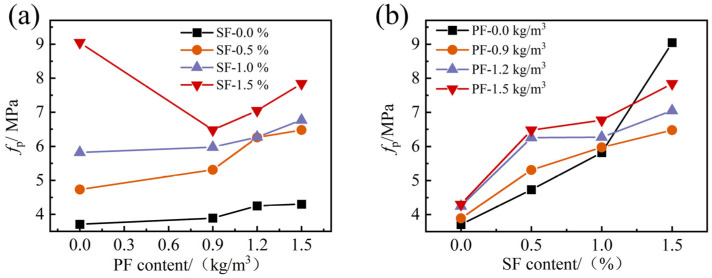
The effect of fiber content on the flexural strength of FRAC: (**a**) PF; (**b**) SF.

The variations in flexural strength in response to PF and SF content are shown in Figure 19. The incorporation of both PF and SF could improve the FRAC flexural strength to different degrees, but SF was more effective. The flexural strength enhancement of single PF- and SF-reinforced FRAC ranged from 4.9 to 16.0% and 27.5 to 217.0%, and the best flexural strength reinforcement effect was achieved when the dosage of PF was 1.5 kg/m^3^ and the SF content reached 1.5%, respectively.

In the HFRFRAC mixtures, the change rule of peak flexural strength was obvious, and the changing trend of the data was similar in each group. When the SF content or PF content was fixed, the peak flexural strength of the FRAC increased with the increase in PF content or SF content. It can be seen that the highest peak flexural strength occurred in the PF15SF15 group, reaching 7.84 MPa, with an increase of 113% compared to ordinary FRAC. It is worth noting that the peak flexural strength of mixtures with hybrid fiber was lower than the mixtures with single fiber when the SF content was 1.5%, contrary to the situation where the SF content ≤ 1.5%, indicating that the addition of PF reduced the bending moment resistance of SF to some extent, and the larger the SF content, the more obvious the reduction effect.

#### 3.4.2. Residual Flexural Strength

The residual flexural strength is an important index of the flexural performance of fiber-reinforced FRAC that can reflect the load-holding capacity of the specimen after flexural failure. According to ASTM C1609 [42], the residual flexural strength corresponding to net deflections of l/100 (3.0 mm) and l/150 (2.0 mm) is used to evaluate the load-holding capacity after the failure of concrete. While the peak-load deflections of the hybrid fiber mixtures were generally greater than 0.5 mm in this paper, the flexural strength at l/100 (3.0 mm) and l/150 (2.0 mm) was selected to evaluate the load-holding capacity of FRAC after damage.

**Figure 20:**
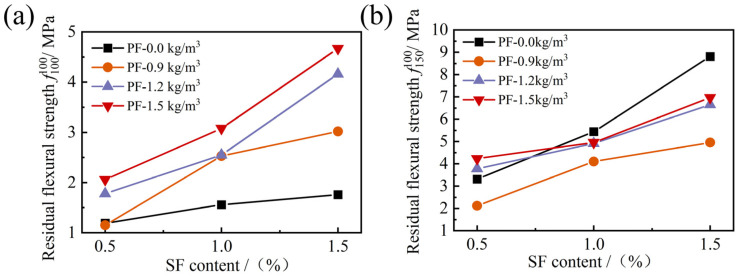
Effect of hybrid fiber on flexural residual strength of FRAC: (**a**) $f_{100}^{100}$; (**b**) $f_{150}^{100}$.

As can be seen in Figure 20, when the PF content was unchanged, the increase in SF content remarkably increased the residual flexural strength, and $f_{100}^{100}$ and $f_{150}^{100}$ increased by more than 0.5 MPa as the SF dosage rose by 0.5%, indicating that the FRAC with high SF content had a solid load-holding capacity after flexural damage. When the SF content was fixed, $f_{100}^{100}$ increased monotonically with the increase in PF content, but $f_{150}^{100}$ did not always increase with the increase in PF content. From Figure 20b, it can be seen that the addition of PF inhibited the enhancement effect of SF on the flexural performance of FRAC with high SF content to some extent and weakened the load-holding capacity of the specimen after flexural failure. As the PF dosage continued to increase, the residual strength also increased, but it is overall less than that of the single SF-reinforced FRAC under the same SF dosage.

#### 3.4.3. Load–Deflection Curve

**Figure 21:**
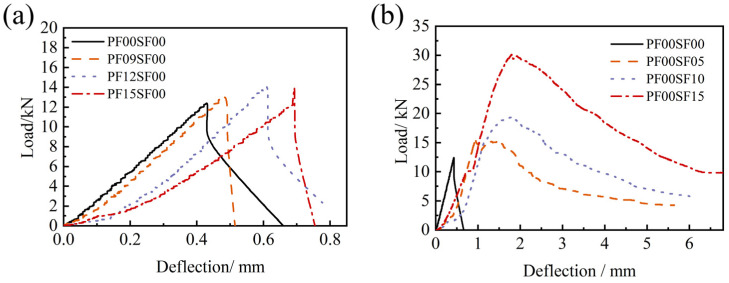
Flexural load–deflection curve of FRAC with single fiber: (**a**) PF; (**b**) SF.

Figure 21 shows the flexural load–deflection comparison curves of the PFRFRAC and SFRFRAC specimens with the ordinary FRAC specimens, respectively. Due to the poor toughness of ordinary FRAC, brittle failure occurred without warning after the peak load, and instability failure occurred quickly after the initial crack of the specimen, and it no longer bore the load. In Figure 21a, as the PF content increased, the curve overall shifted to the right, and the peak deflection increased. The load–deflection curve of the FRAC with single PF added was similar to that of the ordinary FRAC specimen, with the load decreasing rapidly after the peak load was reached, at which time the specimen also cracked into two parts with almost no ductility. For the FRAC with single SF, it can still bear the load after initial crack, and the curve decreased and tended to flatten after the peak load. Furthermore, the slope of its curve is smaller in the failure stage (descending section) compared with that of the ordinary FRAC. The phenomenon that the load hardly decreased or even continued to rise within a certain change of deflection occurred, which suggested that the addition of SF notably improved the toughness of the FRAC. However, there was an apparent difference in the load–deflection curve of the FRAC with different SF dosages. As the SF dosages increased, the overall curve shifted upward, the peak deflection increased, and the bridging and reinforcement effects of SF increased. During the failure stage, the curve became fuller, and after the deflection exceeded 5 mm, it approached the horizontal.

**Figure 22:**
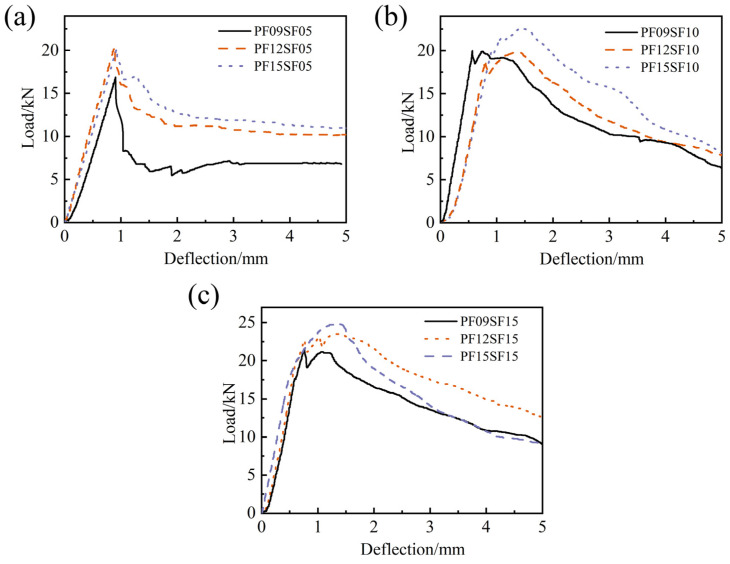
Flexural load–deflection curve of HFRFRAC with different SF contents: (**a**) SF-0.5%; (**b**) SF-1.0%; (**c**) SF-1.5%.

Figure 22 shows the flexural load–deflection curves of FRAC specimens with different PF contents at SF dosages of 0.5%, 1.0%, and 1.5%. It can be seen that at the same SF content, the slope of the ascending part of the curves does not differ much, and the peak deflection changes slightly. With the increase in SF content, the curve became more stable during the failure stage, and the fluctuation phenomenon caused by the secondary strengthening of the load gradually decreased. In Figure 22a,b, the curve showed an overall upward trend with the increase in PF dosage, which is fuller and has a larger peak load compared with that of the SFRFRAC at the same dosage. The difference between the three curves in Figure 22c was not obvious, and the slope of the ascending part was almost the same, but the peak load and area of the curves were remarkably reduced. This indicated that the high SF volume fraction of 1.5% reduced the maximum load that the FRAC bore under bending moments, while further evidence is needed as to whether it reduced the toughness of the FRAC.

#### 3.4.4. Flexural Toughness

The flexural–tensile failure of concrete materials under bending state can comprehensively simulate the force state of materials in actual engineering applications, while the flexural toughness index can reflect the energy consumption of concrete under bending and can also reflect the toughening effect of fiber on concrete. During the test, both the ordinary FRAC and PFRFRAC showed brittle failure after the peak load, indicating that the single PF incorporated had no remarkable enhancement effect on the toughness of the FRAC, while the FRAC with added SF could still bear the load after the peak load, and its toughness was significantly enhanced. Therefore, the next step was mainly to analyze the flexural toughness of the FRAC with steel fiber added.

**Figure 23:**
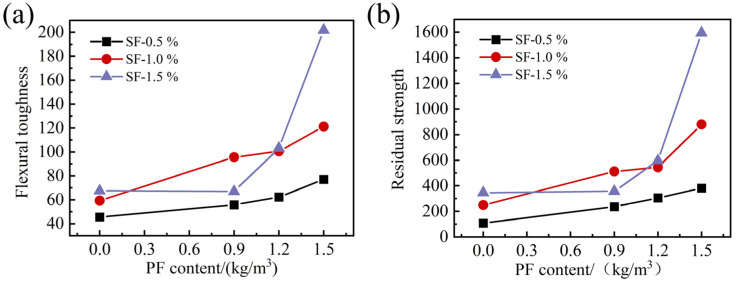
Variation of flexural performance indexes with fiber dosage: (**a**) $I_{20}$; (**b**) $R_{10,20}$.

In Table 10, due to the fact that the peak deflection of the FRAC mixed with SF was much greater than the first crack deflection, and the difference in load curves was not obvious when the deflection was between 3$\delta_{1}$ and 5.5$\delta_{1}$, the calculated values of $I_{5}$, $I_{10}$, and $R_{5,10}$ for each group were relatively close, without any apparent pattern. Only $I_{20}$ and $R_{10,20}$ could well reflect the energy consumption near the end of the test. The variations in $I_{20}$ and $R_{10,20}$ in response to fiber content are shown in Figure 23.

As can be seen in Figure 23a, the flexural toughness index $I_{20}$ of the FRAC with added SF increased with the increase in PF content, and the toughness index of the hybrid fiber mixtures was higher than that of the single-doped SF mixtures. $I_{20}$ reached the highest value of 201.84 in the PF15SF15 group. When the volume fraction of SF was 0.5% and 1.0%, the trend and increment of $I_{20}$ with PF content were similar, and $I_{20}$ doubled as the PF content increased from 0 to 1.5 kg/m^3^. As for the $I_{20}$ of the specimen with the SF volume fraction up to 1.5%, its growth rate increased with the increase in PF content. Specifically, when the PF content increased from 0 to 0.9 kg/m^3^, the value of $I_{20}$ was almost unchanged. When the PF content increased from 0.9 kg/m^3^ to 1.2 kg/m^3^, the increase in $I_{20}$ was 54.3%, and as the PF content increased from 1.2 kg/m^3^ to 1.5 kg/m^3^, the increase in $I_{20}$ reached 94.8%. On the other hand, when the content of PF was constant, the $I_{20}$ of these groups increased with the increase in SF content, except for the PF09SF15 group. In Figure 23b, the variation ruler of $R_{10,20}$ and $I_{20}$ was similar, and the residual strength $R_{10,20}$ of the FRAC with added SF increased with the increase in PF content. The $R_{10,20}$ value of the PF15SF15 group was the highest and reached 1594.4, which was remarkably increased by 361.2% compared to the PF00SF15 group with the same SF content.

In summary, the addition of PF alone did not significantly enhance the toughness of FRAC, while steel fiber had a more notable effect on the toughness. However, when PF was mixed with SF, the increase in the content of PF resulted in a great improvement in the FRAC’s toughness. Overall, the toughness of the FRAC was enhanced with the increase in the total volume fraction of fiber, and the flexural toughness was the greatest when the content of PF was 1.5 kg/m^3^ and the volume fraction of SF was 1.5%.

## 4. Conclusions

In this study, compressive, splitting tensile, and flexural tests were carried out on 16 groups of fully recycled aggregate concrete (wherein both natural coarse and fine aggregates are completely replaced by recycled aggregate) to investigate the influence of polypropylene fiber, steel fiber, and hybrid steel–polypropylene fiber on the mechanical properties of FRAC. The main conclusions are as follows.

The impact of polypropylene fiber on the slump value of FRAC was relatively small, but steel fiber had a significant negative effect on the slump. And the addition of single SF and hybrid steel–polypropylene fiber reduced the slump by approximately 64.7–85.5% and 67.2–89.6%, respectively, compared with that of ordinary FRAC.

Both the compressive strength and splitting tensile strength of the FRAC reinforced with steel fiber or polypropylene fiber were significantly improved, and the combination of PF and SF was more effective than that of single-doped fiber. Moreover, the best increment of the compressive strength of recycled concrete was achieved when the PF dosage was 1.2 kg/m^3^ and the SF volume fraction was 0.5%, with an increase of 56.9% compared to ordinary FRAC.

SF played a leading role in the splitting tensile strength and cracking resistance of the FRAC for hybrid fiber mixtures. When the PF dosage was unchanged, an increase in SF dosage significantly enhanced the splitting tensile strength. The splitting tensile strength was the highest among the same SF dosage when the PF dosage was 0.9 kg/m^3^, and when the SF volume fraction was 0.5, 1%, and 1.5%, the increase compared to ordinary FRAC was 42.1%, 59.5%, and 113.3%, respectively.

Compared with PF, the increased SF content could more effectively enhance the crack resistance of FRAC under tensile stress and improve the splitting tensile toughness. While in hybrid fiber groups, the peak toughness and residual toughness of FRAC decreased with the increase in PF content when the SF dosage was unchanged, when the PF dosage was 0.9 kg/m^3^ and the SF volume fraction was 1.5%, T_1_ and T_2_ had the maximum values of 53.98 N·m and 223.62 N·m, respectively.

The peak flexural strength increased with the increase in SF or PF content, and the peak flexural strength of SFRFRAC was much larger than that of PFRFRAC. When 1.5% SF was added alone, the peak flexural strength of the FRAC had a maximum increase of 217.0%. Similarly, the addition of SF alone in the FRAC is more effective compared to that of PF in terms of improving the flexural toughness. Although there is no obvious enhancement in FRAC toughness when polypropylene fiber was added alone, the increase in PF dosage resulted in a great improvement in FRAC toughness when PF was combined with SF.

Overall, the flexural toughness and residual strength of FRAC showed an increasing trend with the increase in total fiber dosage. The maximum values of flexural toughness and residual strength were obtained when the PF dosage was 1.5 kg/m^3^ and the SF volume fraction was 1.5%.

## Figures and Tables

**Table 1:** Performance index of cement.

Index	Specific Surface Area (m^2^/kg)	Setting Time (min)		Loss on Ignition (%)	Compressive Strength (MPa)		Flexural Strength (MPa)	
		Initial Setting	Final Setting		3 d	28 d	3 d	28 d
Test results	345	230	280	2.81	23.5	44.8	5.6	8.5

**Table 2:** Performance indexes of fly ash.

Index	Fineness Modulus	Bulk Density (kg/m^3^)	Moisture Content (%)	SO_3_ (%)	Water Demand Ratio
Test result	5.692	1120	0.10	0.933	92

**Table 3:** Chemical composition of cement and fly ash.

Components (%)	SiO_2_	CaO	Al_2_O_3_	Fe_2_O_3_	MgO	SO_3_	K_2_O	TiO_2_
Cement	27.73	46.31	13.54	3.09	3.09	2.82	0.984	0.688
Fly ash	54.74	3.36	33.33	2.30	0.867	0.933	2.21	1.01

**Table 4:** Gradation of fine aggregate.

Sieve size (mm)	4.75	2.36	1.18	0.6	0.3	0.15
Cumulative sieve residue (%)	2	35	54	71	81	92

**Table 5:** Performance indexes of RCA.

Index	Apparent Density (kg/m^3^)	Crushing Index	Water Absorption Rate (%)	Mud Content (%)
Test result	2612	16.4	6.95	0.4

**Table 6:** Gradation of RCA.

Sieve size (mm)	2.36	4.75	9.5	16	19	26.5
Cumulative sieve residue (%)	98.42	95.57	69.14	49.31	9.88	0

**Table 7:** Properties of investigated fiber.

Fiber Types	Length (mm)	Diameter (mm)	Density (kg/m^3^)	Elastic Modulus (GPa)	Tensile Strength (MPa)
Steel fiber	35	0.75	7800	200	380
Polypropylene fiber	9	0.05	900	4.0	400

**Table 8:** Mix proportion design in the test (kg/m^3^).

Mixture	Specimen No.	Water	Cement	Fly Ash	Sand	RCA	SP	PF	SF
Ordinary FRAC	PF00SF00	200	457	114	628	942	5.484	0	0
PFRFRAC	PF09SF00	200	457	114	628	942	5.484	0.9	0
	PF12SF00	200	457	114	628	942	5.484	1.2	0
	PF15SF00	200	457	114	628	942	5.484	1.5	0
SFRFRAC	PF00SF05	200	457	114	623	935	5.484	0	39
	PF00SF10	200	457	114	618	927	5.484	0	78
	PF00SF15	200	457	114	613	920	5.484	0	117
HFRFRAC	PF09SF05	200	457	114	623	935	5.484	0.9	39
	PF12SF05	200	457	114	623	935	5.484	1.2	39
	PF15SF05	200	457	114	623	935	5.484	1.5	39
	PF09SF10	200	457	114	618	927	5.484	0.9	78
	PF12SF10	200	457	114	618	927	5.484	1.2	78
	PF15SF10	200	457	114	618	927	5.484	1.5	78
	PF09SF15	200	457	114	613	920	5.484	0.9	117
	PF12SF15	200	457	114	613	920	5.484	1.2	117
	PF15SF15	200	457	114	613	920	5.484	1.5	117

Note: PF09, PF12, and PF15 mean that polypropylene fiber was incorporated into recycled concrete at 0.9 kg/m^3^, 1.2 kg/m^3^, and 1.5 kg/m^3^, respectively; SF05, SF10, and SF15 mean that steel fiber was mixed into recycled concrete at 0.5%, 1.0%, and 1.5% by volume, respectively.

**Table 9:** Average and standard deviation of flexural performance test results of fiber FRAC.

Specimen No.	$f_{1}$ /MPa	$f_{p}$ /MPa	$\delta_{1}$ /mm	$\delta_{p}$ /mm	$f_{100}^{100}$ /MPa	$f_{150}^{100}$ /MPa
PF00SF00	-	3.71 ± 0.26	-	0.43 ± 0.03	-	-
PF09SF00	-	3.89 ± 0.05	-	0.48 ± 0.05	-	-
PF12SF00	-	4.25 ± 0.22	-	0.61 ± 0.03	-	-
PF15SF00	-	4.30 ± 0.32	-	0.69 ± 0.01	-	-
PF00SF05	2.22 ± 0.12	4.73 ± 0.17	0.30 ± 0.02	0.95 ± 0.03	1.19 ± 0.08	3.31 ± 0.10
PF00SF10	2.83 ± 0.31	5.82 ± 0.41	0.51 ± 0.07	1.70 ± 0.08	1.56 ± 0.03	5.44 ± 0.06
PF00SF15	3.96 ± 0.20	9.05 ± 0.23	0.49 ± 0.06	1.74 ± 0.11	1.76 ± 0.06	8.81 ± 0.09
PF09SF05	2.52 ± 0.08	5.31 ± 0.06	0.37 ± 0.02	1.15 ± 0.04	1.15 ± 0.08	2.12 ± 0.07
PF12SF05	3.02 ± 0.18	6.26 ± 0.21	0.35 ± 0.03	1.13 ± 0.03	1.78 ± 0.09	3.78 ± 0.17
PF15SF05	2.98 ± 0.10	6.48 ± 0.16	0.37 ± 0.04	1.17 ± 0.04	2.06 ± 0.15	4.23 ± 0.10
PF09SF10	3.00 ± 0.13	5.97 ± 0.13	0.18 ± 0.02	0.85 ± 0.06	2.53 ± 0.04	4.10 ± 0.26
PF12SF10	2.82 ± 0.15	6.27 ± 0.38	0.26 ± 0.04	1.34 ± 0.11	2.55 ± 0.03	4.91 ± 0.04
PF15SF10	2.90 ± 0.18	6.77 ± 0.21	0.30 ± 0.03	1.41 ± 0.03	3.08 ± 0.11	5.86 ± 0.07
PF09SF15	3.40 ± 0.16	6.48 ± 0.16	0.19 ± 0.01	1.12 ± 0.01	3.02 ± 0.09	4.95 ± 0.19
PF12SF15	2.66 ± 0.17	7.05 ± 0.22	0.25 ± 0.01	1.42 ± 0.06	4.16 ± 0.11	6.65 ± 0.11
PF15SF15	3.19 ± 0.20	7.84 ± 0.25	0.29 ± 0.02	1.61 ± 0.09	4.67 ± 0.08	6.96 ± 0.34

Note: (-) indicates that the value does not exist.

**Table 10:** Flexural toughness indexes of fiber-reinforced FRAC.

Specimen No.	$I_{5}$	$I_{10}$	$I_{20}$	$R_{5,10}$	$R_{10,20}$
PF00SF05	9.25	34.66	45.43	508.2	107.7
PF00SF10	9.84	34.35	59.32	490.2	249.7
PF00SF15	11.92	32.88	67.45	419.2	345.7
PF09SF05	14.67	32.19	55.85	350.4	236.6
PF12SF05	12.21	31.60	62.13	387.8	305.3
PF15SF05	14.00	38.82	76.92	496.4	381.0
PF09SF10	13.17	44.54	95.69	627.4	511.5
PF12SF10	13.55	46.13	100.53	651.6	544.0
PF15SF10	13.79	33.30	121.23	390.2	879.3
PF09SF15	12.63	31.23	66.88	372.0	356.5
PF12SF15	12.10	43.63	103.20	630.6	595.7
PF15SF15	13.46	42.40	201.84	578.8	1594.4

## Data Availability

No new data were created or analyzed in this study. Data sharing is not applicable to this article.
